# Assessment of frailty in elderly patients attending a multidisciplinary wound care centre: a cohort study

**DOI:** 10.1186/s12877-021-02676-y

**Published:** 2021-12-18

**Authors:** Mariona Espaulella-Ferrer, Joan Espaulella-Panicot, Rosa Noell-Boix, Marta Casals-Zorita, Marta Ferrer-Sola, Emma Puigoriol-Juvanteny, Marta Cullell-Dalmau, Marta Otero-Viñas

**Affiliations:** 1 Tissue Repair and Regeneration Laboratory (TR2Lab), Centre for Health and Social Care Research (CESS), University of Vic – Central University of Catalonia (UVIC-UCC), Fundació Hospital Universitari de la Santa Creu de Vic, and Hospital Universitari de Vic, 08500 Vic, Barcelona Spain; 2 Hospital Universitari de la Santa Creu de Vic, 08500 Vic, Barcelona Spain; 3 Central Catalonia Chronicity Research Group (C3RG), Fundació Hospital Universitari de la Santa Creu de Vic, and Hospital Universitari de Vic, 08500 Vic, Barcelona Spain; 4 Research group on Methodology, Methods, Models and Outcomes of Health and Social Sciences (M3O), Faculty of Health Sciences and Welfare, Centre for Health and Social Care Research (CESS), University of Vic-Central University of Catalonia (UVIC-UCC), C. Sagrada Família, 7, Barcelona 08500 Vic, Spain; 5 Hospital Universitari de Vic, 08500 Vic, Barcelona Spain; 6 Quantitative BioImaging (QuBI) Lab, University of Vic – Central University of Catalonia (UVIC-UCC), 08500 Vic, Barcelona Spain; 7 Faculty of Sciences and Technology, University of Vic – Central University of Catalonia (UVIC-UCC), C. de la Laura, 13, 08500 Vic, Barcelona Spain

**Keywords:** Frailty, Elderly, Wound healing, Non-healing wounds, Wound care centre

## Abstract

**Background:**

The incidence of frailty and non-healing wounds increases with patients’ age.

Knowledge of the relationship between frailty and wound healing progress is greatly lacking.

**Methods:**

The aim of this study is to characterize the degree of frailty in elderly patients attending a multidisciplinary wound care centres (MWCC). Additionally, we seek to assess the impact of frailty on the wound healing rate and wound healing time. An open cohort study was conducted on 51 consecutive patients aged > 70 years treated for wounds at an MWCC of an intermediate care hospital. The frailty score was determined according to the Frail-VIG index. Data were collected through patient questionnaires at the beginning of the study, and at 6 months or upon wound healing. Wounds were followed up every 2 weeks. To analyse the relationship between two variables was used the Chi-square test and Student’s or the ANOVA model. The t-test for paired data was used to analyse the evolution of the frailty index during follow-up.

**Results:**

A total of 51 consecutive patients were included (aged 81.1 ± 6.1 years). Frailty prevalence was 74.5% according to the Frail-VIG index (47.1% mildly frail, 19.6% moderately frail, and 7.8% severely frail). Wounds healed in 69.6% of cases at 6 months. The frailty index (FI) was higher in patients with non-healing wounds in comparison with patients with healing wounds (IF 0.31 ± 0.15 vs IF 0.24 ± 0.11, *p* = 0.043). A strong correlation between FI and wound healing results was observed in patients with non-venous ulcers (FI 0.37 ± 0.13 vs FI 0.27 ± 0.10, *p* = 0.015). However, no correlation was observed in patients with venous ulcers (FI 0.17 ± 0.09 vs FI 0.19 ± 0.09, *p* = 0.637). Wound healing rate is statically significantly higher in non-frail patients (8.9% wound reduction/day, P25-P75 3.34–18.3%/day;AQ6 *p* = 0.044) in comparison with frail patients (3.26% wound reduction/day, P25-P75 0.8–8.8%/day).

**Conclusion:**

Frailty is prevalent in elderly patients treated at an MWCC. Frailty degree is correlated with wound healing results and wound healing time.

## Background

Aging is frequently associated with multimorbidity, and along with multiple diseases the occurrence of non-healing ulcers is relevant [1]. Most non-healing wounds are associated with some of the most common conditions among older patients, such as vascular disease, venous insufficiency, disability, unrelieved pressure, and diabetes [1–3]. Patients suffering from non-healing wounds are mostly in the aging population presenting with multimorbidity [3]. Tissue repair capacity worsens with age and wound healing has been described as being reduced in older patients (> 70 years) in comparison with younger ones [4]. Slower healing increases the risk of infection and the likelihood of the wound becoming chronic [5]. These factors lead to more complex wound management in the elderly.

Multidisciplinary wound care centres (MWCC) have emerged to care for patients with complex wounds that require specific advanced therapies due to large wound size, delayed healing time, complex aetiology, and patient systemic disease [6]. These centres are staffed by professionals from different disciplines, trained in devising individualized therapeutic plans and guarantying care continuity. In comparison with other levels of care, MWCC have been shown to decrease healing time and improve the patient care experience [7–9]. MWCC care for a variety of non-healing wounds, the main therapeutic strategies focus rather on the treatment of local wound factors, however the introduction of comprehensive assessment could help in considering the patient as a whole. The implication of local factors (desiccation, infection, maceration, necrosis, pressure) in wound healing progression has been studied in depth [10]. However, it is less known how the general factors act in healing, i.e., type of treatment, systemic disease, multimorbidity, age, etc. [11]. Given patients’ local and systemic condition, it becomes essential to characterize the profiles of older patients to ensure proper management of non-healing wounds in the context of multidisciplinary wound units.

Patient multimorbidity, the presence of two or more chronic conditions, and frailty which represent a global syndrome of decreased physiologic reserve, lead to increased vulnerability to adverse health outcomes [12, 13]. Additionally, multimorbidity increases the likelihood of being frail by around twofold [14]. When multimorbidity is associated with frailty, a special situation is created where the proposed care model is based on situational diagnosis, shared decision-making, and designing an individualized therapeutic plan [15, 16]. Frailty evaluation is currently used as a tool for determining healthcare for complex patients and to assist in decision-making [15, 17, 18]. Multimorbidity is currently the most prevalent chronic disorder, also confirmed in elderly patients with non-healing wounds, that poses a challenge for the management of these patients.

Globally, there are two types of instruments for assessing frailty: frailty phenotype instruments and deficit accumulation indexes. Frailty phenotype instruments, which are based on the Fried model [19, 20] measure physical parameters. The Fried model is mostly used in situations of disability prevention and scores robust to frail patients. An example of this model is the SHARE-Frailty Instrument (SHARE-FI) [21]. On the other hand, the model of deficit accumulation is focus on frailty indexes. Frailty indexes evaluate co-morbidities, functional and cognitive decline, social factors, and geriatric syndromes. The more conditions patients present, the higher the frailty score [15, 22]. Frailty indexes are used as a clinical decision-making instrument. An example of this instrument is Frail-VIG index (“VIG” is the Spanish/Catalan abbreviation for Comprehensive Geriatric Assessment) [23]. Frailty can be determined by using several tools, in this study we used the Frail-VIG index because it allows a rapid geriatric assessment and we compared it with the SHARE-FI which is well validated in the outpatient population.

We hypothesize that patients’ clinical condition is essential to be considered in addition to wound local wound characteristic determination for a better wounds’ management. In this work, we determined the frailty index in elderly patients requiring complex wound treatment at a regional MWCC for better characterization of this patient population. In addition, we evaluated the correlation between the degree of frailty and wound healing outcomes as a potential clinical marker with a prognostic value for cure that could help professionals in clinical decision-making for wound management.

## Material and methods

### Design and study population

This open cohort study was carried out in a MWCC of an intermediate-level university healthcare hospital in Spain.

**Fig. 1:**
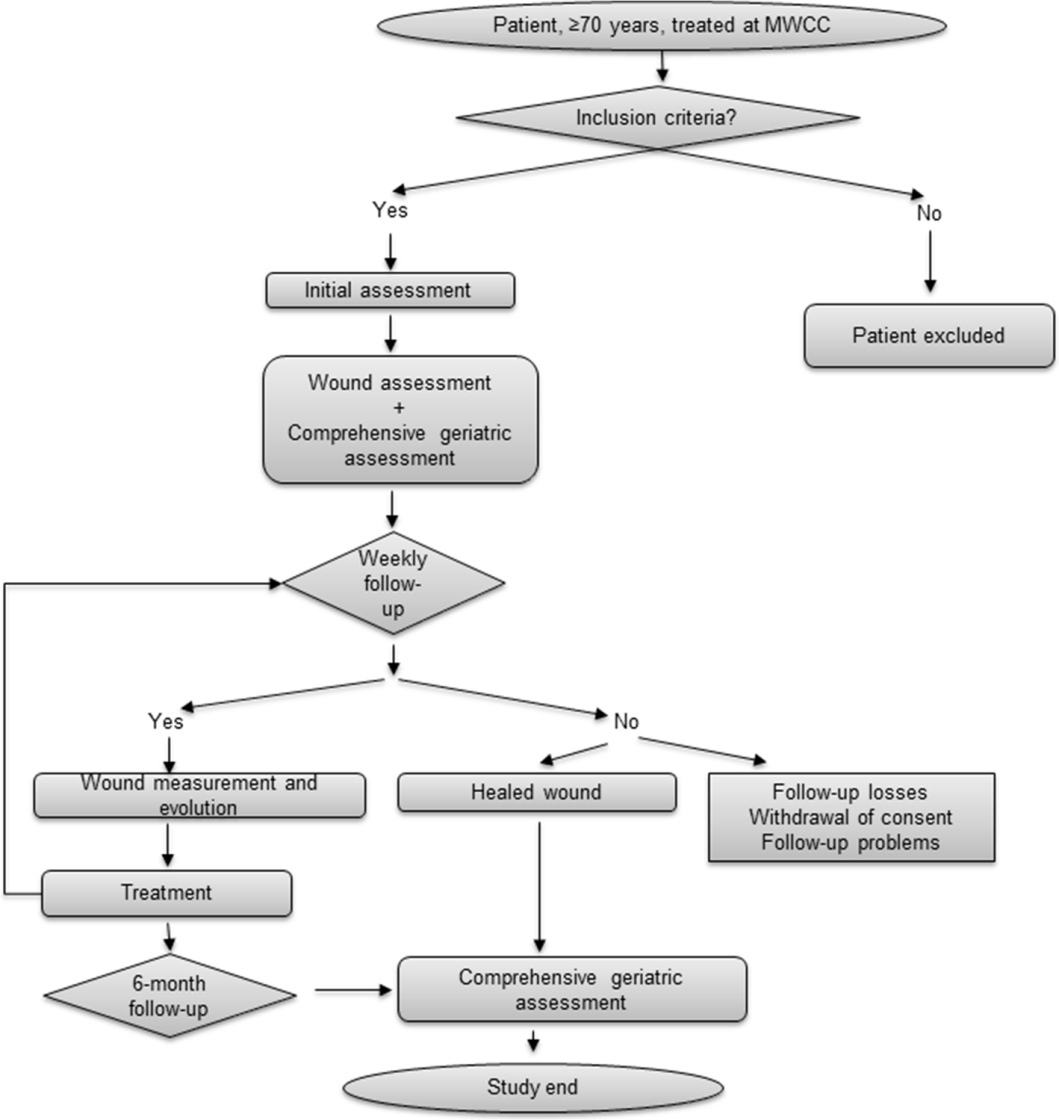
Overview of patients’ follow up

Figure 1 shows an overview of the interventions and assessment of this study conducted between March 2018 and March 2020. All consecutive patients treated in our MWCC that fulfilled the inclusion criteria of being over 70 years old were invited to participate in the study. Exclusion criteria were: clinical follow-up could not be performed; patients did not adhere to the prescribed medical treatments; patients in an imminent end-of-life situation; and patients in a situation of clinical instability due to an acute process. The recruitment process was performed by the nurses who treated the patients’ wounds. At the beginning of the study, patients underwent a comprehensive geriatric assessment, with special emphasis on the frailty index and local wound, according to standard MWCC protocol. Patients follow up was every 2 weeks and until wounds healed or 6 months had passed. Wound size was measured every 2 weeks, and frailty was determined at the beginning of the study and at the end.

A standard clinical protocol was used to evaluate the patient’s wounds and to determine the cause of the wound by an aetiological diagnosis. Wounds management was performed according the current institutional clinical guidelines and were applied depending of the wounds’ aetiology.

### Outcome measures

Patients’ frailty score was determined through two methodologies: the Frail-VIG index and the SHARE-FI instrument. The Frail-VIG index [23], based on comprehensive geriatric assessment, includes 25 items that evaluate functionality, cognition, social status, geriatric syndromes, and comorbidities. The Frail-VIG index allows classifying patients into four groups according to the Frail-VIG score: 0–0.19 = non-frail, 0.20–0.35 = mildly frail; 0.36–0.49 = moderately frail; and ≥ 0.50 = severely frail. The SHARE-FI instrument [24] is based on a phenotypic approach with some modifications. SHARE-FI evaluates five adapted phenotypic frailty items: grip strength and four self-reported items: fatigue, loss of appetite and/or eating less than usual, difficulty in climbing stairs and/or walking 100 m, and low level of physical activity. SHARE-FI application classifies patients into non-frail, pre-frail, and frail.

The outcome measures of our study were:

- sociodemographic data: age and gender.
- likelihood of cognitive impairment using Mini-cog [25]: patients who score 0–2 have a high likelihood of cognitive impairment, while those scoring 3–5 have a low probability.
- functionality: evaluating basic daily living activities using the Barthel index [26], and instrumental daily living activities asking three questions concerning whether the patient is able to handle money, handle medications, and use the telephone [27, 28].
- comorbidities associated to wound healing: hypertension, type 2 diabetes mellitus, dyslipidaemia, obesity, venous insufficiency, and peripheral artery diseases [29, 30].
- social situation: living at home (alone or with relatives), living in a nursing home.
- nutritional status: using the mini nutritional assessment (MNA) test [31], ≥ 24 identifies patients with good nutritional status, a score between 17 and 23.5 identifies patients at risk of malnutrition, and a score < 17 identifies patients with protein-calorie malnutrition.
- gait speed: evaluated by 10-m walk test (10MWT) [32], with a cutoff value for poor physical performance of ≤0.8 m/s.
- wound aetiology: clinical term that describes the cause of the wound (venous ulcers: chronic venous insufficiency, arterial ulcers: deficit in blood supply, etc.).
-  wound age: period between the wound’s appearance and the incorporation of the patient in the study.
-  wound size: measured using a Clinicgram@ device that uses computer vision techniques to determine wound area through wounds images [33].
- healed wound: when wound size was reduced by ≥95%.
- healing rate: evaluated by applying a mathematical model to a minimum of three wound area measurements [34]. Time series corresponding to surface area measurements of the same wound were collected at different patient visits.
- health service delivery: during the follow-up we reviewed the number of health interventions received by each patient (medical visits, admissions, local wound complications).

### Statistical analysis

Data obtained were analysed using the SPSS Statics software version 26.0. Quantitative variables that followed the normal distribution were expressed as mean and standard deviations (SD). For qualitative variables, absolute frequencies and percentages were calculated. For the analysis of the relationship between two qualitative variables, the Chi-square test was used (or the Fisher test in 2 × 2 tables when the expected frequencies were less than five) and Student’s or the ANOVA model were used to analyse the relationship between quantitative and qualitative variables. The t-test for paired data was used to analyse the evolution of the frailty index during follow-up. *P*-values lower than 0.05 were considered statistically significant.

## Results

### Characteristics of the study population

A total of 51 consecutive patients aged > 70 years attending the MWCC between March 2018 and March 2020 were included. The mean age was 81.1 ± 6.2 years. Females accounted for 64.7% (*n* = 33). Of all participants, 58.8% (*n* = 30) lived with relatives and 37.2% (*n* = 19) lived alone. Patients’ functional status analysis showed a mean Barthel index [26] of 82.2 ± 17.7, which corresponds to mild dependency.

When analysing comorbidities, 49.0% (*n* = 25) of patients had ≥3 disorders associated with the development of non-healing wounds, where the most prevalent diseases were hypertension 78.4% (*n* = 40), venous insufficiency 52.9% (*n* = 27), obesity 41.2% (*n* = 21) and type 2 diabetes mellitus 37.2% (*n* = 19). Nutritional status analysis, via the mini nutritional assessment (MNA) test, determined that 7.8% (n = 4) of them presented malnutrition (Table 1).

**Table 1:** Patient characteristics

Patient Characteristics	Results n (%)
Gender	
Female	33 (64.7%)
Male	18 (35.3%)
Age (years), mean ± SD	81.1 years ±6.2
Functionality	
Basic activities of daily living (Barthel index)	82.2 ± 17.7
Need help for instrumental activities of daily living:	
Using the phone	6 (11.8%)
Handling finances	23 (45.1%)
Handling medication	16 (31.4%)
Cognitive status	
Dementia diagnoses	6 (11.7%)
Mini-cog:	
Score > 3 (low likelihood of cognitive impairment)	23 (45.1%)
Score ≤ 2 (high likelihood of cognitive impairment)	28 (54.9%)
Comorbidities associated with wounds	
1	11 (21.6%)
2	15 (29.4%)
≥ 3	25 (49.0%)
Nutritional status	
MNA test:	
Normal nutritional status	36 (70.6%)
At risk of malnutrition	11 (21.6%)
Malnourished	4 (7.8%)
Serum albumin concentration (g/dl)	3.7 ± 0.4
Total cholesterol (mg/dl)	185.0 ± 63.7
Gait Speed	
≤ 0.8 m/s	36 (70.5%)
> 0.8 m/s	15 (29.4%)

The 51 patients included in the study presented a total of 66 wounds. The most common aetiology was venous ulcers (36.36% *n* = 25). The wounds had a median (P_25_-P_75_) of 8.1 (3.6–22.7 cm^2^) and 51.51% (*n* = 34) were recurrent wounds. Wounds had been present for < 3 months in 48.4% (*n* = 32), 3–6 months in 30.3% (*n* = 20), and > 6 months in 21.2% (*n* = 14) of cases (Table 2).

**Table 2:** Wound characteristics

Wound Characteristics	Results n (%)
Aetiology	
Venous	25 (36.3%)
Traumatic	9 (19.6%)
Arterial	9 (13.6%)
Diabetic	5 (7.5%)
Others	15 (22.7%)
Recurrent wounds included	34 (51.5%)
Wound size (cm^2^), median (P_25_-P_75_)	8.1 (3.6–22.7 cm^2^)
Classification according to wound age	
> 6 months	14 (21.2%)
Between 3 and 6 months	20 (30.3%)
< 3 months	32 (48.4%)

### Patient frailty assessment

Frailty evaluation using the Frail-VIG index showed that 25.5% (*n* = 13) of patients were not frail, while the rest of patients presented mild frailty 47.1% (*n* = 24), moderate frailty 19.6% (*n* = 10) or severe frailty 7.8% (*n* = 4). Accordingly, frailty data obtained using the SHARE-FI instrument showed the following: 19.6% (*n* = 10) of patients were non-frail, 23.5% (*n* = 12) of patients were pre-frail, and 56.9% (*n* = 29) of patient were frail (Table 3).

**Table 3:** Frailty score in the cohort study using Frail-VIG index and SHARE-FI instrument

Frailty measurements	Results n (%)
Patient distribution according to Frail-VIG index	
Non-frail	13 (25.5%)
Mild frailty	24 (47.1%)
Moderate frailty	10 (19.6%)
Severe Frailty	4 (7.8%)
Patient distribution according to SHARE-FI	
Non-frail	10 (19.6%)
Pre-frail	12 (23.5%)
Frail	29 (56.9%)

A statistically significant relation of patient frailty classifications was observed when we compared the classifications of the two instruments (*p* < 0.001). Only one out the 51 patients presented a vastly different classification between the two indexes, being classified as frail by the SHARE-FI and non-frail by the Frail-VIG index.

The frailty score determined using the Frail-VIG index showed no statistically significant differences during patient follow-up, from 0.26 ± 0.12 when recruited for the study to 0.23 ± 0.12 at the end of the study (*p* = 0.111).

### Wound evolution

Healing was achieved in 69.6% (*n* = 46) of the wounds in a maximum follow-up time of 6 months. A wound was considered healed when least 95% of its initial area had resolved. Statistically significant differences were observed in wound healing rate between wounds that healed and non-healing wounds (9.5% wound reduction/day vs 1.7% wound reduction/day, respectively, *p* < 0.001).

### Relationship between frailty and wound evolution

The frailty index, according to the Frail-VIG, was higher in patients whose wounds did not heal (mean FI 0.31 ± 0.15 vs FI 0.24 ± 0.11 *p* = 0.043). This difference is even more evident in patients presenting non-venous wounds (FI 0.37 ± 0.13 vs FI 0.27 ± 0.10 *p* = 0.015). However, no correlation was observed between the frailty index and healing rate in patients with wounds of venous aetiology (FI 0.17 ± 0.09 vs FI 0.19 ± 0.09 *p* = 0.637) (Table 4).

**Table 4:** Wound healing correlation with Frail-VIG index score

Wound Characteristics	FRAIL-VIG Score	Frailty Classification	Statistics
Total wounds			
Healing wounds	0.24 ± 0.11	Mildly frail	*p* = 0.043
Non-healing wounds	0.31 ± 0.15	Mildly frail	
Venous ulcers			
Healing wounds	0.19 ± 0.09	Non-frail	*p* = 0.637
Non-healing wounds	0.17 ± 0.09	Non-frail	
Non-venous ulcers			
Healing wounds	0.27 ± 0.10	Mildly frail	*p* = 0.015
Non-healing wounds	0.37 ± 0.13	Moderately frail	

Patients included in the study received 204 medical visits, required 19 admissions, and 14 patients developed clinical complications related to the wound. In addition, three of the study subjects died, two of them as a result of wound complications. Our data demonstrate statistically significant differences between the degree of frailty and use of resources. The frailty index was lower in patients who required fewer medical visits (FI 0.28 ± 0.13 vs FI 0.18 ± 0.12, *p* = 0.009). Similarly, frailer patients required a higher number of admissions (FI 0.38 ± 0.11 vs FI 0.22 ± 0.12 *p* < 0.001) and experienced more wound complications (FI 0.32 ± 0.14 vs FI 0.23 ± 0.12 *p* = 0.021).

Wound healing rate differed between frail patients (3.26% wound reduction/day, P_25_-P_75_ 0.8–8.8%/day) and non-frail patients (8.9% wound reduction/day, P_25_-P_75_ 3.34–18.3%/day; *p* = 0.044).

## Discussion

Our observational study shows the existence of an association between frailty and wound healing. Since one of the main goals of wound clinical units is to shorten healing time, frailty assessment might be introduced in wound management in elderly patients.

Our sample consisted of frail, elderly patients with multimorbidity. In addition, the sample presents some indicators of disability for carrying out basic everyday activities, and most of them required help with at least one of the instrumental activities of daily living, implying a greater functional impact than results reported in the literature for similar populations [28]. A low percentage of patients was diagnosed with dementia (12%) prior to their inclusion in the study. This figure differs greatly from the results obtained in cognitive ability tests, which suggested underdiagnosis of cognitive impairment, in accordance with previously published results [35]. Although, malnutrition negatively influences wound healing [36, 37], in our case, mal- and undernutrition was not relevant and it was not a determining factor in wound healing for our group of patients. This may be because the malnutrition and wound healing strongly correlate with pressure ulcers, which are underrepresented in our patients sample [38, 39]. All this clinical data corroborates that for the patients in this cohort, geriatric assessment detects deficits in several domains highlighting a health vulnerability that goes beyond the wound. In line with previous studies, our data confirm that elderly patients with wounds require a significant degree of healthcare [40].

In the last decade, frailty instruments have been introduced to the regular clinical practice as support for clinicians to achieve better decision-making. Frailty tools had been used to characterize a population, yielding a risk stratification, and having identified patients at greater risk of adverse health outcomes [16, 41–43].

Three-quarters of the patients treated at the MWCC present with frailty. The way patients are identified as being frail, whether through the Frail-VIG index, the SHARE-FI test or physical performance tests such as gait speed, hardly affects their classification, as confirmed by other authors [44, 45]. This figure contrasts with the frailty detected in the community-dwelling population aged > 70 years, where Rivas-Ruiz et al. reported 26% of frailty in community-dwelling elderly persons in Spain using a phenotype tool [46]. Another systematic review, conducted by Collard et al., identifies a very variable spectrum of frailty in community-dwelling older people that ranges from 4.9–59.1% [47]. This large difference in the identification of frailty is most likely related to the fact that patients with non-healing wounds have a high multimorbidity load, some degree of disability, and a high prevalence of mild cognitive impairment.

Our results demonstrated that both instruments (Frail-VIG and SHARE-FI) are able to characterize and classify the population appropriately. So, we have chosen to evaluate patients using the Frail-VIG index as it enables rapid geriatric assessment and the detection of areas of intervention [23].

As the frailty score did not reveal any statistically significant differences during patient follow-up, it suggests that frailty evaluation could be performed at any time of the wound care process, unless clinically relevant issues emerge. Based on our data, we propose frailty assessment at any point of wound follow-up, especially in the event of healing delay or non-healing.

Our data identifies an association between the degree of frailty and wound healing, both evaluated from the variable ‘healing/not healing’ and in relation to the variable ‘healing rate’. Our results suggest that determining the healing rate parameter might prove highly useful for the early prediction of delayed wound healing.

Wound healing is related to widely-known and much studied local factors, and systemic factors [10, 48]. Frailty acts as a systemic factor in wound healing. This idea is strengthened by the observation of different behaviour in relation to frailty between wounds of venous aetiology and others. This data is in accordance with the fact that local factors have a major impact on venous ulcers and they are less influenced by a systemic issue as degree of frailty [10, 48]. So, for venous ulcers management it might be more important to guarantee the patients’ engagement to compression therapy than to modify the therapeutic plan according to the patients’ frailty status. In contrast, in wounds of other aetiologies (arterial, DM2, etc.), the degree of frailty correlates very well with wound healing capacity, which makes sense because such wound aetiologies correlate with systemic diseases [49]. Our data confirms the frame of frailty, describing that frailer patients tend to have poorer health outcomes.

In accordance with other medical and surgical areas in which the assessment of frailty is used to identify patients prone to poor health outcomes [50, 51], our results suggest that establishing frailty may prove useful for wound healing management according to the relation of frailty with healing delay and/or absence of healing. A greater number of advanced therapeutic strategies are available for the treatment of non-healing wounds. However, the effectiveness of these new therapies is not clear. Our results show that a frailty index is a good prognostic indicator of wound healing that could be used for clinical decision-making to improve treatment, not only according to local wound factors, but also patients’ global health status [52]. This study demonstrates that establishing healing rate may also have a prognostic value, in line with data from previous studies [53].

MWCC are usually integrated by a multidisciplinary team that allows not only wound care treatment based on wound aetiology but also according to patients’ global needs. Our results suggested that because of the high prevalence of frailty in patients treated at our MWCC, it would be useful to include the measurement of frailty as part of the regular assessment of patients in wound units.

Consideration and evaluation of frailty are extremely important components in caring for the growing number of elderly patients with complex wounds. While the study of frailty in relationship to wound healing is in its infancy, our results reveal that there is enough data to begin to unravel the complexities associated with caring for frail elderly individuals with complex wounds. Further research is needed both to improve our understanding and our treatment strategies for this particularly frail and at-risk population.

Our study has some limitations, we have a low number of participants, because only those patients who could be assured of follow-up for the next 6 months were included. In addition, the patients included in the study presented different type of wounds aetiology. So, our study allows to demonstrate an association between the presence of frailty and wound healing, however in any case it had been established a causal relationship.

Our data suggest that classification according to different degrees of frailty could help in wound management in elderly patients. In our opinion, patients with severe frailty and non-healing wounds could benefit from a palliative approach, however, patients with moderate/mild frailty might be candidates for advanced wound therapies.

## Conclusions

We describe for the first time that frailty in patients treated at an MWCC is highly prevalent. Degree of frailty is correlated with wound healing and healing rate. However, this relationship is not clear in patients with venous ulcers. Based on our data, we propose including frailty assessment as a routine practice in old patients with non-healing wounds to achieve a more personalized clinical approach. Further studies, including a greater number of patients, are needed in order to fully understand how frailty affects the healing response.

## Data Availability

The datasets used and analysed during the current study are available from the corresponding author on reasonable request.

## Abbreviations

10MWT: 10-Meter Walk Test
FI: Frailty Index
GDS: Global Deterioration Scale
MNA: Mini Nutritional Assessment
MWCC: Multidisciplinary Wound Care Centres
SD: Standard Deviation
SHARE-FI: Survey of Health, Ageing and Retirement in Europe - Frailty Index

## References

[1] Gould LJ, Abadir P, Brem H, et al. Chronic wound repair and healing in older adults: current status and future research. Wound Repair Regen. 2015;23:1–13. 10.1111/wrr.12245. 10.1111/wrr.12245 PMC4414710 25486905

[2] Gist S, Tio-Matos I, Falzgraf S, Cameron S, Beebe M (2009). Wound care in the geriatric client. Clin Interv Aging.

[3] Erfurt-Berge C, Renner R (2015). Chronic wounds – recommendations for diagnostics and therapy. Rev Vasc Med.

[4] Wicke C, Bachinger A, Coerper S, Beckert S, Witte MB, Königsrainer A (2009). Aging influences wound healing in patients with chronic lower extremity wounds treated in a specialized wound care center. Wound Repair Regen.

[5] Keyes BE, Liu S, Asare A (2016). Impaired epidermal to dendritic T cell signaling slows wound repair in aged skin article impaired epidermal to dendritic T cell signaling slows wound repair in aged skin. Cell.

[6] Gottrup F (2004). A specialized wound-healing center concept: importance of a multidisciplinary department structure and surgical treatment facilities in the treatment of chronic wounds. Am J Surg.

[7] Gottrup F, Pokorná A, Bjerregaard J, Vuagnat H (2018). Wound centres—how do we obtain high quality? The EWMA wound centre endorsement project. J Wound Care.

[8] Kim P, Evans K, Steinberg J, Pollard M, Attinger C (2013). Critical elements to building an effective wound care center. J Vasc Surg.

[9] de Leon J, Bohn G, DiDomenico L, Fearmonti R, Gottlieb H (2016). Wound care centers:critical thinking and treatment strategies for wounds. Wounds.

[10] Hess CT (2011). Checklist for factors affecting wound healing. Adv Skin Wound Care.

[11] Han G, Ceilley R (2017). Chronic wound Healing : a review of current management and treatments. Adv Ther.

[12] Rockwood K (2005). What would make a definition of frailty successful?. Age Ageing.

[13] Clegg A, Young J, Iliff S, Rikkert MO, Rockwood K (2013). Frailty in elderly people. Lancet.

[14] Vetrano DL, Palmer K, Marengoni A, Marzetti E, Lattanzio F, Roller-Wirnsberger R, Samaniego LL, Rodríguez-Mañas L, Bernabei R, Onder G (2019). Frailty and multimorbidity: a systematic review and meta-analysis. J Gerontol - Ser A Biol Sci Med Sci.

[15] Walston J, Buta B, Xue Q-L (2018). Frailty screening and intervntions: considertaions for clinical practice. Clin Geriatr Med.

[16] Amblàs-Novellas J, Espaulella-Panicot J, Rexach L, Fontecha B, Inzitari M, Blay C, Gómez-Batiste X (2015). Frailty, severity, progression and shared decision-making: a pragmatic framework for the challenge of clinical complexity at the end of life. Eur Geriatr Med.

[17] Cesari M, Prince M, Thiyagarajan J (2016). Frailty: an emerging public health priority. J Am Med Dir Assoc.

[18] Khatry K, Peel NM, Gray LC, Hubbard RE (2018). The utility of the frailty index in clinical decision making. J Frailty Aging.

[19] Fried LP, Ferrucci L, Darer J, Williamson JD, Anderson G (2004). Untangling the concepts of disability, frailty, and comorbidity: implications for improved targeting and care. J Gerontol - Ser A Biol Sci Med Sci.

[20] Fried L, Tangen C, Walston J (2001). Frailty in older adults: evidence for a phenotype. J Gerontol A Biol Sci Med Sci.

[21] Romero-ortuno R (2013). The SHARE frailty instrument for primary care predicts mortality similarly to a frailty index based on comprehensive geriatric assessment. Geriatr Gerontol Int.

[22] Walston JD, Bandeen-Roche K (2015). Frailty: a tale of two concepts. BMC Med.

[23] Amblàs-Novellas J, Martori JC, Espaulella J, Oller R, Molist-Brunet N, Inzitari M, Romero-Ortuno R (2018). Frail-VIG index: a concise frailty evaluation tool for rapid geriatric assessment. BMC Geriatr.

[24] Romero-Ortuno R, Walsh CD, Lawlor BA, Kenny RA (2010). A frailty instrument for primary care: findings from the survey of health, ageing and retirement in Europe (SHARE). BMC Geriatr.

[25] Borson S, Scanlan JM, Watanabe J, Tu SP, Lessig M (2005). Simplifying detection of cognitive impairment: comparison of the Mini-Cog and Mini-mental state examination in a multiethnic sample. J Am Geriatr Soc.

[26] Sainsbury A, Seebass G, Bansal A, Young JB (2005). Reliability of the Barthel index when used with older people. Age Ageing.

[27] Lawton MP, Brody EM (1949). Assessment of older people: self-maintaining and instrumental activities of daily living. J Am Med Assoc.

[28] Arnau A, Espaulella J, Serrarols M, Canudas J, Formiga F, Ferrer M (2012). Factores asociados al estado funcional en personas de 75 o más años de edad no dependientes. Gac Sanit.

[29] Jockenhöfer F, Gollnick H, Herberger K (2016). Aetiology, comorbidities and cofactors of chronic leg ulcers: retrospective evaluation of 1 000 patients from 10 specialised dermatological wound care centers in Germany. Int Wound J.

[30] Cheung C (2010). Older adults and ulcers: chronic wounds in the geriatric population. Adv Skin Wound Care.

[31] Vellas B, Villars H, Abellan G (2006). Overview of the MNA--its history and challenges. J Nutr Heal Aging.

[32] Amatachaya S, Kwanmongkolthong M, Thongjumroon A, Boonpew NAP, Saensook W, Thaweewannakij T, Hunsawong T (2019). Influence of timing protocols and distance covered on the outcomes of the 10-meter walk test. Physiother Theory Pr feb.

[33] Reifs D, Valls G, Casals M, Reig-Bolaño R (2019). New superpixels for chronic ulcers segmentation. Front Artif Intell Appl.

[34] Cullell-Dalmau M, Otero-Viñas M, Manzo C (2020). Research techniques made simple: deep learning for the classification of dermatological images. J Invest Dermatol.

[35] Amjad H, Roth DL, Sheehan OC, Lyketsos CG, Wolff JL, Samus QM (2018). Underdiagnosis of dementia: an observational study of patterns in diagnosis and awareness in US older adults. J Gen Intern Med.

[36] Palmieri B, Vadalà M, Laurino C (2019). Nutrition in wound healing: investigation of the molecular mechanisms, a narrative review. J Wound Care.

[37] Herberger K, Müller K, Protz K, Zyriax BC, Augustin M, Hagenström K (2020). Nutritional status and quality of nutrition in chronic wound patients. Int Wound J.

[38] Molnar JA, Vlad LG, Gumus T (2016). Nutrition and chronic wounds: improving clinical outcomes. Plast Reconstr Surg.

[39] Horn SD, Fife CE, Smout RJ, Barrett RS, Thomson B (2013). Development of a wound healing index for patients with chronic wounds. Wound Repair Regen.

[40] Sen CK, Gordillo GM, Roy S, Kirsner R, Lambert L, Hunt TK, Gottrup F, Gurtner GC, Longaker MT (2009). Human skin wounds : a major and snowballing threat to public health and the economy. Wound Repair Regen.

[41] Rockwood K, Theou O, Mitnitski A (2015). What are frailty instruments for?. Age Ageing.

[42] Cardona-Morrell M, Lewis E, Suman S, Haywood C, Williams M, Brousseau AA, Greenaway S, Hillman K, Dent E (2017). Recognising older frail patients near the end of life: what next?. Eur J Intern Med.

[43] Shi SM, McCarthy EP, Mitchell SL, Kim DH (2020). Predicting mortality and adverse outcomes: comparing the frailty index to general prognostic indices. J Gen Intern Med.

[44] Pilotto A, Rengo F, Marchionni N, Sancarlo D, Fontana A, Panza F, Ferrucci L (2012). Comparing the prognostic accuracy for all-cause mortality of frailty instruments: a multicentre 1-year follow-up in hospitalized older patients. PLoS One.

[45] Woo J, Leung J, Morley JE (2012). Comparison of frailty indicators based on clinical phenotype and the multiple deficit approach in predicting mortality and physical limitation. J Am Geriatr Soc.

[46] Rivas-Ruiz F, Machón M, Contreras-Fernández E, Vrotsou K, Padilla-Ruiz M, Díez Ruiz AI, de Mesa BY, Vergara I (2019). Prevalence of frailty among community-dwelling elderly persons in Spain and factors associated with it. Eur J Gen Pract.

[47] Collard RM, Boter H, Schoevers RA, Oude Voshaar RC (2012). Prevalence of frailty in community-dwelling older persons: a systematic review. J Am Geriatr Soc.

[48] Guo S, DiPietro LA (2010). Factors affecting wound healing. J Dent Res.

[49] Zheng Y, Ley SH, Hu FB (2018). Global aetiology and epidemiology of type 2 diabetes mellitus and its complications. Nat Rev Endocrinol.

[50] Panayi AC, Orkaby AR, Sakthivel D (2019). Impact of frailty on outcomes in surgical patients: a systematic review and meta-analysis. Am J Surg.

[51] Shinall MC, Arya S, Youk A (2019). Association of preoperative patient frailty and operative stress with postoperative mortality. JAMA Surg.

[52] Hoogendijk EO, Afilalo J, Ensrud KE, Kowal P, Onder G, Fried LP (2019). Frailty: implications for clinical practice and public health. Lancet.

[53] Lavery LA, Barnes SA, Keith MS, Seaman JW, Armstrong DG (2008). Prediction of healing for postoperative diabetic foot wounds based on early wound area progression. Diabetes Care.

